# World Water Day — March 22, 2017

**DOI:** 10.15585/mmwr.mm6610a6

**Published:** 2017-03-17

World Water Day is sponsored by the United Nations and observed each year on March 22. This year, World Water Day focuses on wastewater, which includes sewage, storm water, and discarded water used in the community (*1*). Many developing countries have inadequate wastewater management strategies because they lack resources, infrastructure, available technology, or space. Untreated wastewater in these countries is often disposed of directly into rivers, lakes, or oceans, polluting the environment and increasing the risk for disease transmission (*2*).

The World Health Organization’s Sustainable Development Goal 6 aims in part to improve access to sanitation facilities (*3*), an important first step in proper wastewater management. As of 2015, approximately 2.4 billion persons worldwide lacked improved sanitation facilities (i.e., facilities designed to ensure users will not come into contact with human waste), and 946 million persons practiced open defecation (*4*). In countries that have sanitation facilities, waste streams must be collected and properly treated before being disposed into the environment. Although 68% of the global population now uses an improved sanitation facility, worldwide only 20% of wastewater receives proper treatment (*4*,*5*).

Through the CDC Innovation Fund, CDC is collaborating with Sanivation (http://www.sanivation.com), a startup company, on a novel approach to improving access to sanitation facilities and wastewater management strategies in Kenya. Company representatives install toilet facilities in Kenyan households and make twice-weekly visits to collect waste from toilets. Sanivation treats the collected waste with solar thermal energy and blends it with carbonized agricultural waste to produce low-cost charcoal briquettes for cooking fuel and heating homes.

Additional information about World Water Day is available at http://www.unwater.org/worldwaterday. Additional information about CDC’s initiatives to improve global access to water and sanitation is available at https://www.cdc.gov/healthywater/global.
